# Diagnostic validation of two SARS-CoV-2 immunochromatographic tests in Slovenian and Croatian hospitals

**DOI:** 10.3325/cmj.2021.62.513

**Published:** 2021-10

**Authors:** Martina Ifko, Željka Tkalčić Švabek, Iva Friščić, Mirjana Mariana Kardum Paro, Ingrid Prkačin, Lovorka Đerek, Ana Livun, Miha Skvarč

**Affiliations:** 1 Doba Faculty, Maribor, Slovenia; 2 Clinical Department of Laboratory Diagnostics, University Hospital Center Zagreb, Zagreb, Croatia; 3 Department of Medical Biochemistry and Laboratory Medicine, Merkur University Hospital, Zagreb, Croatia; 4 Department of Internal Medicine, Merkur University Hospital, Zagreb, Croatia; 5 Clinical Department for Laboratory Diagnostics, Dubrava University Hospital, Zagreb, Croatia; 6 Microbiological Department, General Hospital Jesenice, Jesenice, Slovenia

## Abstract

**Aim:**

To diagnostically validate two point-of-care (POC) rapid antigen tests for SARS-CoV-2 by comparing their results with those of laboratory-based real-time polymerase chain reaction tests (RT-PCR).

**Methods:**

The study enrolled 455 patients from two Slovenian and two Croatian hospitals. The NADAL COVID-19 Ag Test (Nal von Minden, Moers, Germany) and ALLTEST COVID-19 Antigen Test (Hangzhou ALLTEST Biotech Co., Ltd, Hangzhou, China) were diagnostically validated in emergency care departments of two Slovenian hospitals, while only ALLTEST COVID-19 Antigen Test was validated in two Croatian hospitals.

**Results:**

The antigen test results were in very good agreement with the RT-PCR results (Cohen's Kappa between 0.747 and 0.891 for the NADAL COVID-19 and between 0.820 and 0.954 for the ALLTEST COVID-19). The NADAL COVID-19 Ag Test had the sensitivity between 66.67% and 92.31%, with a negative predictive value between 85.51% and 99.2%. The ALLTEST COVID-19 Antigen Test had the sensitivity between 81.39% and 91.11%, with a negative predictive value between 85.45% and 98.78%.

**Conclusion:**

The antigen tests are practical and reliable screening assays for SARS CoV-2 in emergency care departments. Both antigen tests can be used as screening tests to reduce the number of patients waiting for RT-PCR results. Even more, they can be used to quickly isolate COVID-19 patients and reduce hospital transmissions.

The COVID-19 pandemic has entered every pore of health care systems worldwide and endangered their functioning. An adequate COVID-19 response should entail timely testing and sufficient testing capacities (1). A major obstacle in the current fight against SARS-CoV-2 is a much greater number of patients compared with the number of real-time polymerase chain reaction (RT-PCR) tests that can be performed. Therefore, we need quick and accurate alternative tests, such as antigen detection tests, to detect the virus presence in respiratory tract samples. Antigen tests provide rapid results and can be used efficiently by trained non-laboratory personnel as point of care tests (POCT). Rapid results may help in stopping the uncontrolled virus spread in hospitals (2). The World Health Organization (WHO) has set the standards for the use of antigen tests in terms of their negative and positive predictive value and acceptable diagnostic accuracy (3). Nevertheless, most of the available antigen tests underwent only emergency use authorizations, with limited data available on clinical validations. In addition, the number of published studies on the diagnostic performance of antigen tests vs RT-PCR is still low. In this article, we present the diagnostic validation data for two POC antigen tests in two Slovenian emergency care departments (General Hospital Jesenice and General Hospital Slovenj Gradec) and two Croatian hospitals (University Hospital Merkur and University Hospital Dubrava, both in Zagreb).

## Patients and methods

In General Hospital Jesenice, 106 patients were tested with the NADAL COVID-19 Ag Test (Nal von Minden, Moers, Germany) and 90 patients with ALLTEST COVID-19 Antigen Test (Hangzhou AllTest Biotech Co., Ltd, Hangzhou, China). All results were compared with those of Seegene Allplex 2019-nCoV test (SeeGene, Seoul, South Korea). In General Hospital Slovenj Gradec, 92 samples were tested with the NADAL COVID-19 Ag Test and 94 with ALLTEST COVID-19 Antigen Test, and the results were compared with those of Cobas 6800 SARSCoV-2 Test (Roche, Indianapolis, IN, USA). University Hospital Merkur and University Hospital Dubrava used only one test, the NADAL COVID-19, for 51 specimens and compared the results with those of SARS-CoV-2 RT-PCR assay (PCRBiosystems Ltd, London, UK), performed according to a published RT-PCR protocol (2).

Diagnostic validation of the NADAL COVID-19 test started in the middle of September 2020. Clinical Hospital Jesenice started using the antigen tests in the middle of October 2020, when the region experienced an uncontrolled disease spread. By the end of October, the 14-day incidence was 1970.8 cases per 100 000 inhabitants, reflecting an increase in positive community cases by 53% per week (4).

The study enrolled patients with at least one clinical sign of COVID-19 (fever above 37.5°C, fatigue, rhinorrhea/rhinitis, dry cough, dyspnea, loss of taste and smell, or gastrointestinal problems with at least one sign of upper respiratory tract disease). Croatian hospitals collected two swabs at the same time, one of which was used for RT-PCR testing in the laboratory and the other for POC antigen assay testing. In the Slovenian hospitals, the antigen test was performed at first for POCT, and the patients were asked to wait for the results. If the result was positive or presumed false negative, a second and contralateral nasopharyngeal swab was collected within an hour of the first sample collection, by the same health care worker, for RT-PCR testing in local public health microbiology laboratories. In Croatian hospitals, all results were compared with RT-PCR tests. Patients who had COVID-19 symptoms for more than five days were excluded as it was advised in the instruction manuals for both tests. Statistical analysis was performed with MedCalc Statistical Software, version 18.11.6 (MedCalc Software bvba, Ostend, Belgium).

The study was approved by the Ethics Committee of General Hospital Jesenice for Slovenian hospitals (2020-02) and by the Ethics Committees of Merkur University Hospital (11593) and Dubrava University Hospital (2020/1012-04). Study participants gave informed written or oral consent.

## Results

In General Hospital Jesenice, the positive percent agreement rate (PPA) and negative percent agreement rate (NPA) of the NADAL COVID-19 Ag Test vs RT-PCR were 66.76% (95% CI 35.88%-90.07%) and 100% (95% CI 96.15%-100%), respectively (Table 1). The overall percent-agreement rate (OPA) was 96.23%. In General Hospital Slovenj Gradec, PPA, NPA, and OPA of the NADAL COVID-19 Ag Test vs RT-PCR were 69.70% (95% CI 51.29%-84.41%), 100% (95% CI 93.93%-100%), and 89.13%, respectively (Table 1). In university hospitals Merkur and Dubrava, PPA, NPA and OPA were 84.61% (95% CI 54.55%-98.08%), 100% (95% CI 90.75%-100%), and 96.08%, respectively (Table 1).

**Table 1:** Comparison of NADAL COVID-19 Ag Test vs a laboratory-based real time-polymerase chain reaction test in two Slovenian and two Croatian hospitals

	General Hospital Jesenice		General Hospital Slovenj Gradec		University hospitals Merkur and Dubrava combined		All hospitals	
	N	vs RT-PCR (%)	N	vs RT-PCR (%)	N	vs RT-PCR (%)	N	vs RT-PCR (%)
PPA (sensitivity)	8/12	66.67 (95% CI 35.88- 90.07)	23/33	69.70 (95% CI 51.29- 84.41)	11/13	84.61 (95% CI 54.55- 98.08)	55/69	79.71 (95% CI 68.31- 88.44)
NPA (specificity)	94/94	100 (95% CI 96.15 - 100)	59/59	100 (95% CI 93.93- 100)	38/38	100 (95% CI 90.75- 100)	122/122	100 (95% CI 97.02- 100)
OPA	102/106	96.23 (95% CI 90.62- 98.96)	82/92	89.13 (95% CI 80.92- 94.66)	49/51	96.08 (95% CI 86.54- 99.52)	177/191	93.42 (95% CI 89.38- 96.27%
Cohen`s Kappa	0.780	n.a.	0.747	n.a.	0.891	n.a.	0.834	n.a.
Prevalence	12 / 106	11.32	33/92	35.87	13/51	25.49	55/191	28.80
PPV	8/8	100	23/23	100	11/11	100	55/55	100
NPV	94/98	95.92 (95% CI 91.62 - 98.96)	59/69	85.51 (95% CI 77.86- 90.83)	38/40	95 (95% CI 84.15- 98.55)	122/136	89.71 (95% CI 86.21- 93.96)

*Abbreviations: PPA – positive percent agreement; NPA – negative percent agreement; OPA – overall percent agreement; PPV – positive predictive value; NPV – negative predictive value; n. a. – not applicable; N – number of tests; CI – confidence interval.

Cohen`s kappa coefficients in all four hospitals were high (0.780, 0.747, and 0.891, respectively), showing a high-to-perfect agreement between the NADAL COVID-19 and RT-PCR according to a scale of Kappa value interpretation (5).

Taken all hospitals together, PPA, NPA, and OPA of the NADAL COVID-19 vs RT-PCR were 79.71% (95% CI 68.31%-88.44%), 100% (95% CI 97.02%-100%), and 93.42%, respectively. Cohen`s kappa across four hospitals was high (0.834), showing a nearly perfect agreement between the NADAL COVID-19 and RT-PCR (5).

In General Hospital Jesenice, PPA and NPA of the ALLTEST COVID-19 vs RT-PCR were 82.98% (95% CI 69.19%-92.35%) and 100% (95% CI 98.10%-100%), respectively (Table 2). The OPA was 96.65%. In General Hospital Slovenj Gradec, PPA, NPA, and OPA were 92.31% (95% CI 63.97%-99.81%), 100% (95% CI 95.55%-100%), and 98.94%, respectively (Table 2).

**Table 2:** Comparison of ALLTEST COVID-19 antigen assay vs a laboratory-based real time-polymerase chain reaction test in two Slovenian hospitals*

	General Hospital Jesenice		General Hospital Slovenj Gradec		General hospitals Jesenice and Slovenj Gradec combined	
	N	vs RT-PCR	N	vs RT-PCR	N	vs RT-PCR
PPA (sensitivity)	39/47	82.98 (95 CI 69.19-92.35)	12/13	92.31 (95% CI 63.97-99.81)	51/60	85.00 (95% CI 73.43-92.90)
NPA (specificity)	192/192	100 (95% CI 98.10- 100)	81/81	100 (95% CI 95.55-100)	273/273	100 (95% CI 98.67-100)
OPA	231/239	96.65 (95% CI 93.51-98.54)	93/94	98.94 (95% CI 94.22-99.97)	324/333	97.30 (95% CI 94.93 98.76)
Cohen`s Kappa	0.887	n.a.	0.954	n.a.	0.903	n.a.
Prevalence	39/239	19.67	13/94	13.83	52/333	18.02
PPV	39/39	100	12/12	100	51/51	100
NPV	192/200	96.00 (95% CI 92.74- 97.83)	122/136	89.71 (95% CI 86.21- 93.96)	273/282	96.8 (95% CI 94.32- 98.23)

*Abbreviations: PPA – positive percent agreement; NPA – negative percent agreement; OPA – overall percent agreement; PPV – positive predictive value; NPV – negative predictive value; n. a. – not applicable; N – number of tests; CI – confidence interval.

Cohen`s kappa coefficients in General Hospital Jesenice and General Hospital Slovenj Gradec for the ALLTEST COVID-19 were 0.887 and 0.954, respectively, showing a high-to-perfect agreement with RT-PCR according to a scale of Kappa value interpretation (5).

Taken both Slovenian hospitals together, PPA, NPA, and OPA of the ALLTEST COVID-19 vs RT-PCR were 100%, 96.81 (95% CI 94.32%-98.23%), and 97.30%, respectively (Table 2). Cohen`s Kappa was high (0.903), showing a nearly perfect agreement between the ALLTEST COVID-19 and RT-PCR (5).

## Discussion

In the fall of 2020, the number of commercially available antigen tests for SARS CoV-2 soared, but only a few have been clinically validated as POC tests. This is the first study in southeastern Europe evaluating the diagnostic accuracy of rapid immunochromatographic antigen tests for SARS CoV-2 on fresh clinical specimens as true POC tests in emergency care departments.

The sensitivity of the NADAL COVID-19 Ag Test vs RT-PCR was between 66.67% and 84.61%, and the specificity was 100% in all test sites. Cohen`s Kappa was high (between 0.78 and 0.891), showing a nearly perfect agreement between the NADAL COVID-19 Ag Test and RT-PCR. The highest sensitivity and specificity were observed in Croatian hospitals. In Slovenia the highest sensitivity and specificity were observed in a hospital with an infectious disease department. This finding could be explained by the fact that this hospital clinically evaluated which patients were eligible for SARS CoV-2 testing under the supervision of an infectious disease specialist. This hospital probably had the best selection of patients for testing based on clinical symptoms and signs, and the samples were only sent to RT-PCR testing when it was needed to resolve false-negative antigen test results or to confirm antigen-positive test results. At the time of testing, Croatia had a low number of positive samples and a lower COVID-19 incidence than Slovenia. In total, the NADAL COVID-19 Ag Test exceeded the minimum sensitivity and specificity of 80% and 97% as recommended by the WHO (3).

The ALLTEST COVID-19 Antigen Test proved to be an assay of choice in the two Slovenian hospitals. The assay had a good sensitivity, very good in General Hospital Slovenj Gradec (92.31%), with an excellent overall NPV of 93.43%. Cohen`s Kappa was high (between 0.887 and 0.954), showing a nearly perfect agreement with RT-PCR. The ALLTEST COVID-19 is a user-friendly assay since it requires only 10 seconds of incubation. Furthermore, the red test line cannot be mistakenly read as a false positive, which increases the test's reliability. Recently published laboratory-based studies from different countries validating more than one antigen assay for SARS-CoV-2 also showed a high specificity level, ranging from 99.5% to 100%, but a wide range of sensitivity, from 0 to 93.9% (6-10). A laboratory-based study showed the sensitivity and specificity of the BD Veritor SARS CoV-2 antigen test (Becton Dickinson, NJ, USA), which uses an analyzer to detect a signal, to be 76.32% (95% CI 59.39%-87.97%) and 99.53% (95% CI 97.01%-99.98%), respectively, in specimens collected from patients 0-7 days after symptom onset (9). A true POCT study performed in the Netherlands and Aruba compared the Panbio COVID-19 Ag Rapid Test (Abbott, Chicago, IL, USA) and 2-gene target RT-PCR in 1575 mildly symptomatic participants (10). In both settings, the assay specificity was 100% (95% CI 99.7%-100%). Test sensitivity was 72.6% (95% CI 64.5%-79.9%) in the Netherlands and 81.0% (95% CI 69.0%-89.8%) in Aruba (10).

Another laboratory-based study performed on nasopharyngeal swabs taken at the emergency care department of two infectious disease centers in Italy assessed the performance of Standard Q COVID-19 Ag assays (SD-Biosensor, Suwon, South Korea) in 185 randomly selected patients. The sensitivity was 72.1%, while the specificity was 100% (11). In a POC study evaluating the clinical performance of the BD Veritor SARS CoV-2 assay in 475 COVID-19 symptomatic adults at a municipal health service center in the Netherlands, the overall clinical specificity was 100% (95% CI 98.9%-100%) and the sensitivity was 80.7% (95% CI 73.2%-86.9%) compared with RT-PCR (12).

Clinically validated POCT SARS CoV-2 antigen tests could be a good screening tool for use in pandemics. The SARS-CoV-2 pandemic highlighted the shortage of experienced staff as a limitation of RT-PCR tests, which created long waiting times for RT-PCR results (11). All four hospitals in our study used the antigen tests successfully for triage and to lower the number of patients waiting for PCR. By testing all the admitted patients, hospitals could also reduce the number of hospital transmissions with efficient rapid isolation of antigen-positive patients not waiting for RT-PCR results.

The current study was limited by our inability to collect cycle thresholds for semiquantitative estimation of viral concentrations in the specimens, which could explain the low sensitivity of the NADAL COVID-19 Ag Test observed in Slovenian hospitals. Healthcare workers who collected specimens and conducted the testing were not equally well trained, which could have contributed to the relatively low sensitivity of both tests in Jesenice.

In conclusion, our results show that both diagnostically validated SARS-CoV-2 antigen tests exceeded the WHO criteria for minimum sensitivity and specificity of 80% and 97%, respectively. Cohen`s Kappa coefficients for the NADAL COVID-19 and ALLTEST COVID-19 tests were 0.834 and 0.903, respectively, showing very good agreement with RT-PCR. We demonstrated that sound clinical judgment with the help of antigen POC test can differentiate true negative from false-negative results.

In our opinion, the ALLTEST COVID-19 Antigen Test has better performance than the NADAL COVID-19 Ag, as shown by a higher Cohen`s Kappa (0.903). In General Hospital Slovenj Gradec, which at the time of testing faced the highest COVID-19 incidence and tested many patients with COVID-19 pneumonia, Cohen`s Kappa was 0.954. As already mentioned, the ALLTEST COVID-19 is a test of choice for both Slovenian hospitals and is also used for mass testing and on the primary health care level.
